# Isolated congenital cholesteatoma of the mastoid process: a case report

**DOI:** 10.1590/S1808-86942012000400024

**Published:** 2015-10-20

**Authors:** Lídio Granato, Carlos Jorge Silva, Hea J Yoo

**Affiliations:** 1 PhD (Associate Professor); 2 PhD (Assisting Professor); 3 Specialist (Assisting Professor)

**Keywords:** cholesteatoma, congenital, hereditary, neonatal diseases and abnormalities

## INTRODUCTION

Congenital (or primitive) cholesteatoma is a benign disease with slow progressive growth that destroys neighboring structures. It is considered an epidermal cyst originating from the remnants of squamous keratinized epithelium. The disease may appear in several regions of the temporal bone such as in the middle ear (most frequent site) as well as in the petrous apex, cerebellopontine cistern, external acoustic meatus and mastoid process. Congenital cholesteatoma of the mastoid process is the rarest form of presentation in the temporal bone.

## OBJECTIVES

The aim of this report was to present clinical and imaging features of a patient with an isolated congenital cholesteatoma of the mastoid process, and to provide a brief review of the literature.

## CASE REPORT

**Figure 1:**
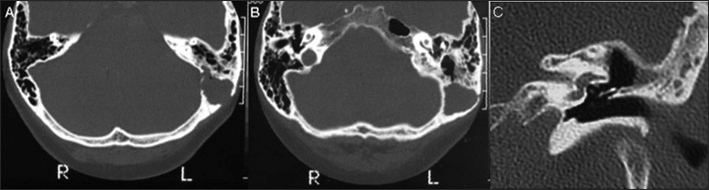
A-B: CT of the temporal bones. Axial images showed a hypoattenuating and expansive lesion in the left mastoid process eroding its medial and lateral walls, adjacent to the left sigmoid sinus. The remaining structures were unremarkable. C: Follow-up coronal CT image of the left temporal bone did not reveal recurrence and showed a large surgical cavity in the mastoid process communicating antrum with tympanic cavity.

A 67-year-old black female from Santos city, São Paulo State, Brazil, had sought medical assistance 5 years earlier for bilateral hearing loss and an 8-year history of pulsatile *tinnitus* in the left ear. She had no history of ear infections or surgery. Physical examination revealed mild pain upon compression of the left mastoid process, and occasional transient dizziness. Both tympanic membranes were normal. Tuning fork and Weber tests were unremarkable. Audiometric evaluation demonstrated bilateral sensorineural hearing loss, particularly for high frequency sounds, with good discrimination. Tympanometry and stapedial reflexes were normal. Brainstem electric response audiometry (BERA) also presented normal results. Computed tomography (CT) scan of the temporal bones showed a hypoattenuating and expansive lesion in the left mastoid process, eroding its medial and lateral walls adjacent to the left sigmoid sinus (Figure 1). The remaining structures were unremarkable. A magnetic resonance (MR) scan of the temporal bones was also performed, showing the lesion was hypointense on T1- and hyperintense on T2-weighted images, with no significant postcontrast enhancement. Based on these results, a left exploratory tympanomastoidectomy was performed under general anesthesia. The cleft of the middle ear was unremarkable. The mastoid cells presented granulation tissue. A large cholesteatoma was identified in the left mastoid process, eroding its medial and lateral walls with exposure of the adjacent dura and sigmoid sinus associated with discrete compression of the homolateral cerebellar hemisphere. The lesion was fully resected, preserving the posterosuperior wall of the external auditory canal. The results of the histological analysis confirmed cholesteatoma. The immediate postoperative period was without complications and the patient reported no further dizziness at follow-up. However, she continues to describe a bilateral hearing deficit and non-pulsatile *tinnitus*. Follow-up CT scan confirmed no recurrence and showed a large surgical cavity in the left mastoid process communicating with the *antrum, aditus ad antrum*, and tympanic cavity. Bone erosion occurred adjacent to the left sigmoid sinus (Figure 1).

## DISCUSSION

In 1953, House^1^ was the first to describe a cholesteatoma behind an intact membrane while in 1991, Proctor^2^ reported that congenital cholesteatoma originated from the same ectoderm which forms a primitive notochord, and that embryonic cell remnants of this ectodermic structure can occur in any cranial bone. In a report by Rashad et al.^3^, Korner published in 1900, these authors stated that congenital cholesteatoma could be diagnosed in the absence of local trauma or infection and in the presence of a normal tympanic membrane. Luntz et al.^4^ in 1997, were the first to describe the imaging features of an isolated congenital cholesteatoma of the mastoid, and defined 3 aspects characteristic of this disease: 1) pain in the upper neck; 2) CT scan revealing a lytic and expansive lesion affecting the mastoid process, without compromise to the middle ear; 3) MR examination revealing hyperintensity on T2-weighted images, with no significant postcontrast enhancement, as seen in our patient. These 3 criteria may not be present in all patients, and other symptoms may coexist in this disease. The rarity of cases may explain the difficulty determining more precise characteristics. Mevio et al.^5^ added instability as a possible symptom of cholesteatoma of the mastoid process. The cholesteatoma in our patient was fully removed. The left sigmoid sinus was exposed but not atelectasic, as described in a similar case with extensive erosion of this region by Mevio et al.^5^ The posterosuperior wall of the external auditory canal was preserved, as described by Luntz et al., despite the possibility of lesion recurrence. The patient was informed of this risk and the need for periodic clinical and imaging follow-up. Some authors have performed surgeries in patients with congenital cholesteatoma of the mastoid that involved destruction of the posterosuperior wall of the external auditory canal, devising a modified radical mastoidectomy. It should be noted however, these patients presented cholesteatoma that had expanded and infiltrated into regions other than the mastoid. Congenital cholesteatoma may appear in various regions of the temporal bone and its clinical manifestation depends on location. Isolated congenital cholesteatoma of the mastoid process is the rarest form of presentation in the temporal bone, with few such cases described in the literature. The only studies found in the literature similar to the present case were conducted by Derlacki & Clemis^6^ in 1965, Luntz et al.^4^ in 1997, and Mevio et al.^5^ in 2002. These authors reported the appearance of cholesteatoma of the mastoid without progression to other regions of the temporal bone, removing all doubts as to their origin. The suspicion that the reported patient had an isolated congenital cholesteatoma of the mastoid process arose when the cleft of the left middle ear, the left tympanic membrane and the left mastoid cells were found to be unremarkable, despite the large lesion in the homolateral mastoid process. The osseous labyrinth was intact and the reported dizziness had no apparent explanation. The pulsatile *tinnutus* may have been due to the lesion's contiguity with the adjacent non-thrombosed left sigmoid sinus. The differential diagnosis of this lesion can be reached based on a finding of cholesterol granuloma. However, cholesterol granulomas are typically hyperintense on T1-weighted images and other rare manifestations include endolymphatic sac tumor and meningiomas.

## CONCLUSION

We described a patient with a rare isolated congenital cholesteatoma of the mastoid process that presented typical clinical and imaging features. The condition, although extremely rare, may be suspected based on clinical and imaging characteristics. However, this suspicion requires subsequent histological confirmation. Management is by surgical treatment with full lesion resection and periodic postoperative follow-up.
